# Long-Term Pollen Monitoring in the Benelux: Evaluation of Allergenic Pollen Levels and Temporal Variations of Pollen Seasons

**DOI:** 10.3389/falgy.2021.676176

**Published:** 2021-07-08

**Authors:** Letty A. de Weger, Nicolas Bruffaerts, Mieke M. J. F. Koenders, Willem W. Verstraeten, Andy W. Delcloo, Pierre Hentges, François Hentges

**Affiliations:** ^1^Department of Pulmonology, Leiden University Medical Center, Leiden, Netherlands; ^2^Department of Public Health and Primary Care, National eHealth Living Lab, Leiden University Medical Center, Leiden, Netherlands; ^3^Mycology and Aerobiology, Sciensano, Brussels, Belgium; ^4^Clinical Chemistry, Elkerliek Hospital, Helmond, Netherlands; ^5^Dispersion and Atmospheric Composition, Royal Meteorological Institute of Belgium, Brussels, Belgium; ^6^Aerobiology Data Analysis Consulting, Centre Hospitalier de Luxembourg, Luxembourg, Luxembourg; ^7^Unit of Immunology-Allergology, Centre Hospitalier de Luxembourg, Luxembourg, Luxembourg

**Keywords:** pollen, seasonal trends, allergy, climate change, Benelux

## Abstract

Airborne pollen is a major cause of allergic rhinitis, affecting between 10 and 30% of the population in Belgium, the Netherlands, and Luxembourg (Benelux). Allergenic pollen is produced by wind pollinating plants and released in relatively low to massive amounts. Current climate changes, in combination with increasing urbanization, are likely to affect the presence of airborne allergenic pollen with respect to exposure intensity, timing as well as duration. Detailed analysis of long-term temporal trends at supranational scale may provide more comprehensive insight into these phenomena. To this end, the Spearman correlation was used to statistically compare the temporal trends in airborne pollen concentration monitored at the aerobiological stations which gathered the longest time-series (30–44 years) in the Benelux with a focus on the allergenic pollen taxa: *Alnus, Corylus, Betula, Fraxinus, Quercus, Platanus*, Poaceae, and *Artemisia*. Most arboreal species showed an overall trend toward an increase in the annual pollen integral and peak values and an overall trend toward an earlier start and end of the pollen season, which for *Betula* resulted in a significant decrease in season length. For the herbaceous species (Poaceae and *Artemisia*), the annual pollen integral and peak values showed a decreasing trend. The season timing of Poaceae showed a trend toward earlier starts and longer seasons in all locations. In all, these results show that temporal variations in pollen levels almost always follow a common trend in the Benelux, suggesting a similar force of climate change-driven factors, especially for *Betula* where a clear positive correlation was found between changes in temperature and pollen release over time. However, some trends were more local-specific indicating the influence of other environmental factors, e.g., the increasing urbanization in the surroundings of these monitoring locations. The dynamics in the observed trends can impact allergic patients by increasing the severity of symptoms, upsetting the habit of timing of the season, complicating diagnosis due to overlapping pollen seasons and the emergence of new symptoms due allergens that were weak at first.

## Introduction

It is widely recognized that the prevalence of respiratory allergies such as asthma and allergic rhinitis is globally increasing, especially in the industrialized world (1, 2). For Belgium, the Netherlands and Luxembourg (whose usual acronym is Benelux) the prevalence of asthma varies between 10 and 15% of the population (3) and for allergic rhinitis it is estimated to be 10–30% (4–6). The increase in prevalence of these diseases may be related to improved hygiene, increased antibiotic use and vaccination, and changes in lifestyle, dietary habit, and air pollution (7). In addition, the increase in amount of airborne allergenic pollen and prolonged pollen seasons caused by climate change and/or by changes in land use may also contribute (8, 9).

According to the Intergovernmental Panel on Climate Change (IPCC) the human influence on the climate system is clear (10), warming of the climate system is unmistakable and globally average temperature has been increasing (11). As an example, the World Meteorological Organization (WMO) reported that 2020 was 1.2 ± 0.1°C warmer than the pre-industrial baseline (1850–1900) (12). These long-term changes in the climate have multiple impacts on the environment and on health.

Pollen emitted by wind pollinating plants is one of the most relevant triggers of respiratory allergies, and therefore monitoring of pollen is relevant for individuals with respiratory allergies. So far, pollen has been monitored for more than five decades in Europe and 25 years ago the first studies appeared noticing changes in airborne pollen concentrations and shifts in the pollen seasons (13–15), Ziello et al. (16) showed in 2012 an increase in the annual pollen count for many pollen taxa in Europe, without a clear relation to temperature change. Since the increase was more pronounced in urban areas than in rural sites, it was suggested that the increase in pollen could be due to a possible anthropogenic rise of atmospheric CO_2_. Several recent studies showed significant increasing airborne pollen concentration trends in relation to the changing climate as well as in shifts in the timing of the pollen seasons in Northern (13, 17, 18) and in southern Europe (19–21). The results often differ per region and pollen type. Most studies show an overall trend of increasing pollen concentrations, in particular for tree species (13, 20), although some other studies present contrasting results (22). Regarding the season timing, shifts also appear to vary depending on the region and the analyzed species, which is illustrated by some examples. Several studies report an advancement of the pollen season of tree species like hazel, birch, oak and pine (17, 18, 23), or of herbaceous plants like grasses and nettles (13). A Swedish study found a season-shift to later end dates for grasses and mugwort (18), while in Belgium both the start and the end of the grass pollen season have shifted to an earlier date (17).

Benelux, which is located in Northwestern Europe, usually refers in many domains to a supranational territory, although it started from a simple economic union. According to the Köppen–Geiger climate classification map for Europe (24), the region has a temperate climate, i.e., no dry season and warm summers, although relatively colder in Luxembourg which lies between 200 and 500 m above sea level. Consequently, Benelux also contains a smooth gradient of biogeographical regions (25) from Atlantic in Netherlands and North Belgium to continental in South-East Belgium and Luxembourg, which are reported to be sensitive to the variations in climate (26). Within this temperate climate region, the average temperature has increased ~2°C since the beginning of the 20th century. The annual rainfall has increased due to more winter precipitation, which contrasts with a decrease in summertime precipitation (27, 28). Five pollen monitoring stations in this region are among the pioneer ones worldwide, so far collecting long time series of airborne pollen concentration data which are valuable for studying potential trends. In this study, we perform a trend analysis of pollen seasons over 39–44 years at two stations in Belgium and two stations in the Netherlands, and over 30 years at one station in Luxembourg. The annual pollen integrals, the peak values in daily concentration and the timing of pollen seasons observed in the past decades are analyzed for a selection of taxa that are clinically considered as moderately relevant (ash, plane, oak, and mugwort) to highly relevant (hazel, alder, birch, and grasses) in regard to pollen induced respiratory allergies in this region.

## Materials and Methods

### Study Sites

**Figure 1:**
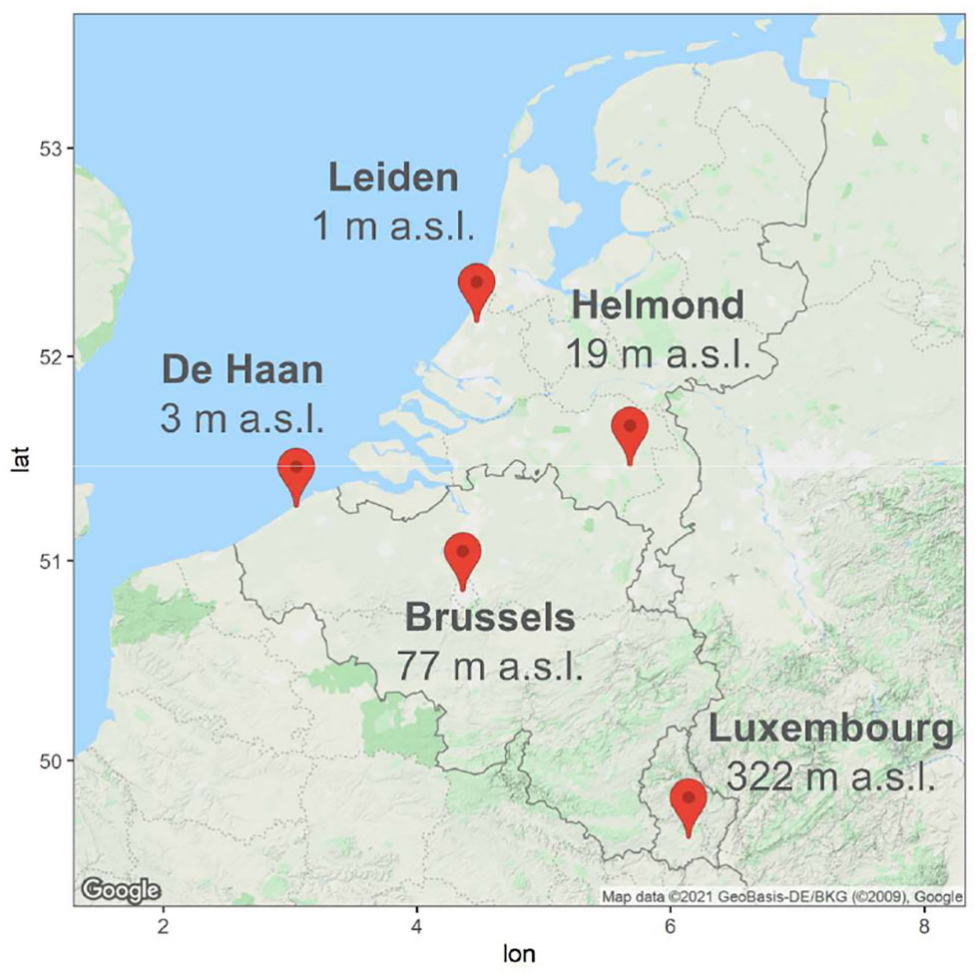
Map of the selected monitoring stations. Corresponding city name and altitude above sea level (a.s.l.) expressed in meters.

The five monitoring sites are located in the Northwestern part of Europe, bordering the North Sea in the Benelux countries (Figure 1). In the center of Belgium, the monitoring site in Brussels Capital Region (16,138 ha, 1,218,255 inhabitants in 2020, while ~960,000 in 2000) is located within a functional urban area that extends to a buffer zone of 30 km around the site (29). The availability of open land surface in this region has been drastically reduced for three decades [– 56% agricultural land, – 45% meadow, and – 40% wasteland (Belgian Federal Statistics 2017)]. While in dominantly rural Belgian areas the allergenic tree species distribution is mainly driven by soil characteristics, in dominantly urban areas it appears to be driven by more environmental covariates explaining urban heterogeneity (29). In particular, inventory datasets of urban green spaces in Brussels show that among the 10 most frequent tree species, a series of them present allergenic properties, i.e., ashes, hornbeams, horse chestnuts, beeches, planes, limes, oaks and birches (30). The sampler is located on the rooftop of a 15 m high building at the Sciensano site in the municipality of Elsene.

The monitoring site located in the coastal Belgian municipality of De Haan (4,614 ha, 12,700 inhabitants in 2020) is surrounded by mixed rural and urban landscapes (29). The airborne pollen levels are usually lower on the Belgian coast than inland, which makes it a recommended location for allergic sufferers during the high pollen season. The sampler is located on the rooftop (10 m above ground level) of the Zeepreventorium building.

Helmond is located in the south center of the Netherlands enclosed by populated towns and suburbs. Helmond has 92,400 inhabitants and the township spans 5,475 ha. The city is densely populated with 1,759 households per square kilometer. Since 1996, the population has grown with 23%. To accommodate this new population, houses were built from this year on, resulting that ~20% of the houses in Helmond date from after 2000. In between, the urbanized areas there are areas of farmland, forest and heather. The streets in the city are lined with oak, linden, alder, birch, maple horse chestnut, and poplar. The sampler is located on the roof of the hospital (~20 m above ground level). In 2000, a large stretch of wasteland close to the hospital was used for building a residential area. Until today, this residential area has still been expanding. More to the north in the Netherlands, Leiden (1 m above sea level) is located at 20 km distance from the coast. The sampler is located on the roof of the hospital (~20 m above ground level) in the middle of the urban area. Leiden has 125,000 inhabitants and the township covers an area of 2,327 ha. Although the city is situated in the most populated part of the Netherlands, to the east the surroundings consist of polderlands. The streets in the city are lined with alder, birch, ash, plane, willow, maple, horse chestnut, oak, or poplar (31). From 1991 to 2000 close to the sampler a stretch of waste land was present where old hospital buildings had been demolished. Since 2000 this waste land has been rebuilt with a large research- and an education building which both were finished in 2006.

Luxembourg City is the capital of the Grand Duchy Luxembourg, and is the highest location above sea level of the five sites. It lies in the southern part of a large plateau of early Jurassic sandstone formation. Luxembourg City has 125,000 inhabitants with a population increase of 30% over the last 30 years. It covers an area of 51.46 km^2^. While 20% of the territory of Luxembourg city is covered by woodland, this even increases to 33% in the surrounding region where the rest of the unbuilt land is mainly covered by grassland. Two third of the trees are deciduous. Among them beech and oak are largely predominant the rest being made up by hornbeam, birch, ash, maple, alder, hazel, willow and poplar, while planes mainly planted on line roads. The sampler is located on the roof of the Centre Hospitalier de Luxembourg about 20 m above ground. The hospital itself is situated in the Western outskirts of the city.

### Pollen Monitoring and Weather Parameters

At all sites, airborne pollen is sampled by using a Hirst-type 7-day volumetric spore trap (32) (Burkard Manufacturing Co., Ltd., UK) placed on rooftops at 10–20 m height above ground level. The methodological guidelines requirements of the European Aerobiology Society (EAS) were followed to the best ability (33), apart from the average daily pollen concentrations (pollen/m^3^) which were calculated after counting two (Brussels, De Haan, and Luxembourg) or three (Leiden, Helmond) longitudinal lines of daily slides. The sampling and the manual identification and counting of pollen by microscopy has been carried out by independent teams in all the stations. Based on abundance and allergenic potential, the following eight taxa were chosen for further analysis: alder (*Alnus* spp.), hazel (*Corylus* spp.), birch (*Betula* spp.), ash, (*Fraxinus* spp.), oak (*Quercus* spp.), plane (*Platanus* spp.), grasses (Poaceae), and mugwort (*Artemisia* spp.).

Weather parameters related to temperature (mean/minimum/maximum of daily temperatures in °C; net solar radiation in J/m^2^ or converted to Watt/m^2^ per day) were obtained from weather stations close to the traps: Ukkel for Brussels, Zeebrugge for De Haan, Schiphol for Leiden; Eindhoven for Helmond. For Luxembourg, weather data were obtained from the Copernicus ERA5 dataset for map coordinates 49.618999N, 6.1E, corresponding to the location closest to the pollen sampler.

### Data Analysis

Since for most sites the data were recorded from January 1st to September 30th, the time series analysis was generally encompassing this monitoring period. Any major data gap of more than 5 days during the usual window of a specific pollen season, involved the omission of the related yearly dataset for a given taxon. *Platanus* was not registered in Leiden before 1982 and in Helmond not before 1986. Due to too low amounts of *Platanus* pollen in De Haan in the early decades the timing of the pollen season was not analyzed for this pollen type at De Haan. The overview of the years used for this study is given in Supplementary Table 1.

The total taxon-specific airborne pollen accumulated during a yearly monitoring period was expressed as Annual Pollen Integral (API) (32). The peak value corresponds to the maximum daily pollen concentration measured during a yearly monitoring period. The peak day is the day on which this maximum was reached, expressed as day number since January 1st. Since a “gold standard” for season start/end dates is lacking we chose two methods based on the percentage of the API, as these methods may be beneficial when API values are low (34). Individual pollen taxa at some of the sites showed low API values (e.g., *Artemisia* in Luxembourg, *Corylus* in De Haan). For the start/end dates of the pollen season, two definitions were used: (i) the day number since January 1st upon which the accumulated sum of daily pollen/m^3^ reached, respectively 1 and 99% of the API, or (ii) respectively 5 and 95%. The length of the season was calculated by subtracting the start day from the end day of the season.

Since most data were not normally distributed according to the Shapiro–Wilk test, trends were analyzed using the non-parametric Spearman rank correlation test between the years and either one of the pollen parameters (API, peak, peak day, start, end, and length of the pollen season) for each site using STATA 14.1 software. Bubble maps showing Spearman Rho coefficient values were generated with RStudio (RStudio Team 2020, MA, USA). To visualize the trends of the yearly parameters in boxplots, the pollen data were averaged per decade: 1981–1990, 1991–2000, 2001–2010, and 2011–2020. A one-way ANOVA with Bonferroni *post-hoc* comparison was conducted to compare the airborne pollen of the sequential decades of each monitoring site (STATA 14.1).

The association between pollen trends and weather trends followed the methodology described by Bruffaerts et al. (13) using R (www.r-project.org) and IDL (www.ittvis.com/idl/). For this analysis we chose three major allergenic taxa that flower in different periods of the year: *Alnus, Betula*, and Poaceae. Briefly, Sen slopes were calculated for each day of the pollen season, grouped by day of the year. This was followed by LOESS smoothing of slopes (fraction of 0.1) of the in-season days. For the association between pollen trends and weather trends, Sen slopes of each day of the year (January–September) were calculated for weather variables followed by LOESS smoothing (fraction 0.1). The association of pollen and weather trends was then determined as the Spearman correlation of pairs of Sen slopes representing the pollen trend and the weather trend for the same in-season pollen day.

## Results

**Figure 2:**
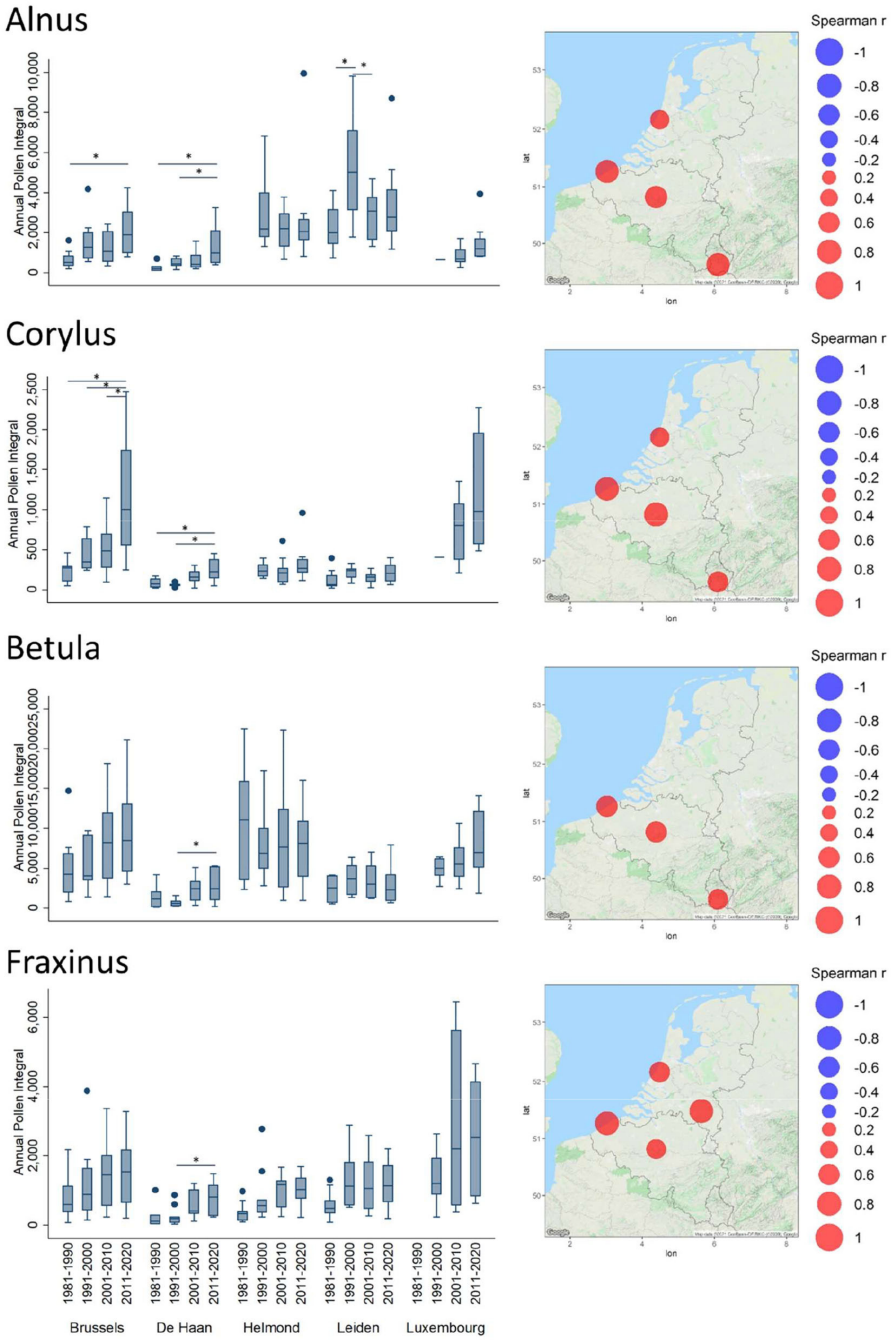
Trends of Annual Pollen Integrals (API) for *Alnus, Corylus, Betula*, and *Fraxinus*. Spearman correlation coefficient (Rho) values between API and years are plotted in bubble maps at the location of the monitoring stations, from negative (in blue) to positive (in red). The bubble size relates to the magnitude of the Rho value. Non-significant values are not shown on the maps. API values, averaged per decade (1981–1990, 1991–2000, 2001–2010, and 2011–2020), are depicted in boxplots. The upper and lower limits of the boxplot are the 75 (Q3) and 25 (Q1) % percentile. The median is shown as a line in the box. The whiskers of a boxplot extend to values known as adjacent values. These are the values in the data that are furthest away from the median on either side of the box but are still within a distance of 1.5 times the interquartile range [=1.5^*^ (Q3–Q1)]. Values outside the adjacent values are plotted as separate points. The horizontal lines above the boxplots indicate significant differences (one-way ANOVA) between the decades; ^*^p < 0.05. Absence of the horizontal lines above box plots indicates differences among those decades are non-significant.

In terms of intensity, all the pollen taxa show clear trends that may be observed either with Spearman correlation values between API and the years (Supplementary Table 2), or with the API values averaged per decade (Figure 2).

For the two early blooming tree taxa, *Alnus* and *Corylus*, significant increasing trends were observed in Brussels, De Haan, Leiden, and Luxembourg. The absolute API values were higher in the North for *Alnus* (Leiden and Helmond) and higher in the South for *Corylus* (Brussels and Luxembourg). The decadal increasing trends appear to be clear when the *Alnus* API started from low values in the first decades to higher values in the last decade (Brussels, De Haan, and Luxembourg); this decadal increase from the first to the last decade was significant for Brussels and De Haan. The already high exposure to *Alnus* pollen in Helmond has not change significantly. The significant increase in the decade 1991–2000 compared to the neighboring decades in Leiden cannot be explained. In contrast, *Corylus* API was in the same range of values in all Benelux stations during the first decades (Figure 2). Over the time, this API has increased significantly in Brussels and Luxembourg, especially during the last decade.

Regarding *Betula* pollen, it is interesting to see that lower API values were observed on the Belgian and Dutch coasts (De Haan and Leiden). The Spearman correlation coefficients (Supplementary Table 2) show significant increasing trends in airborne birch pollen levels in Belgium and Luxembourg. In <4 decades, the mean API values observed in Brussels have reached the same range as in Helmond, where historically they have always been relatively high (Figure 2).

For *Fraxinus*, Spearman correlations were significantly positive in all stations (Figure 2), except in Luxembourg where it was weaker. However, the latter shows a large although not significant trend of increase in API over the decades.

**Figure 3:**
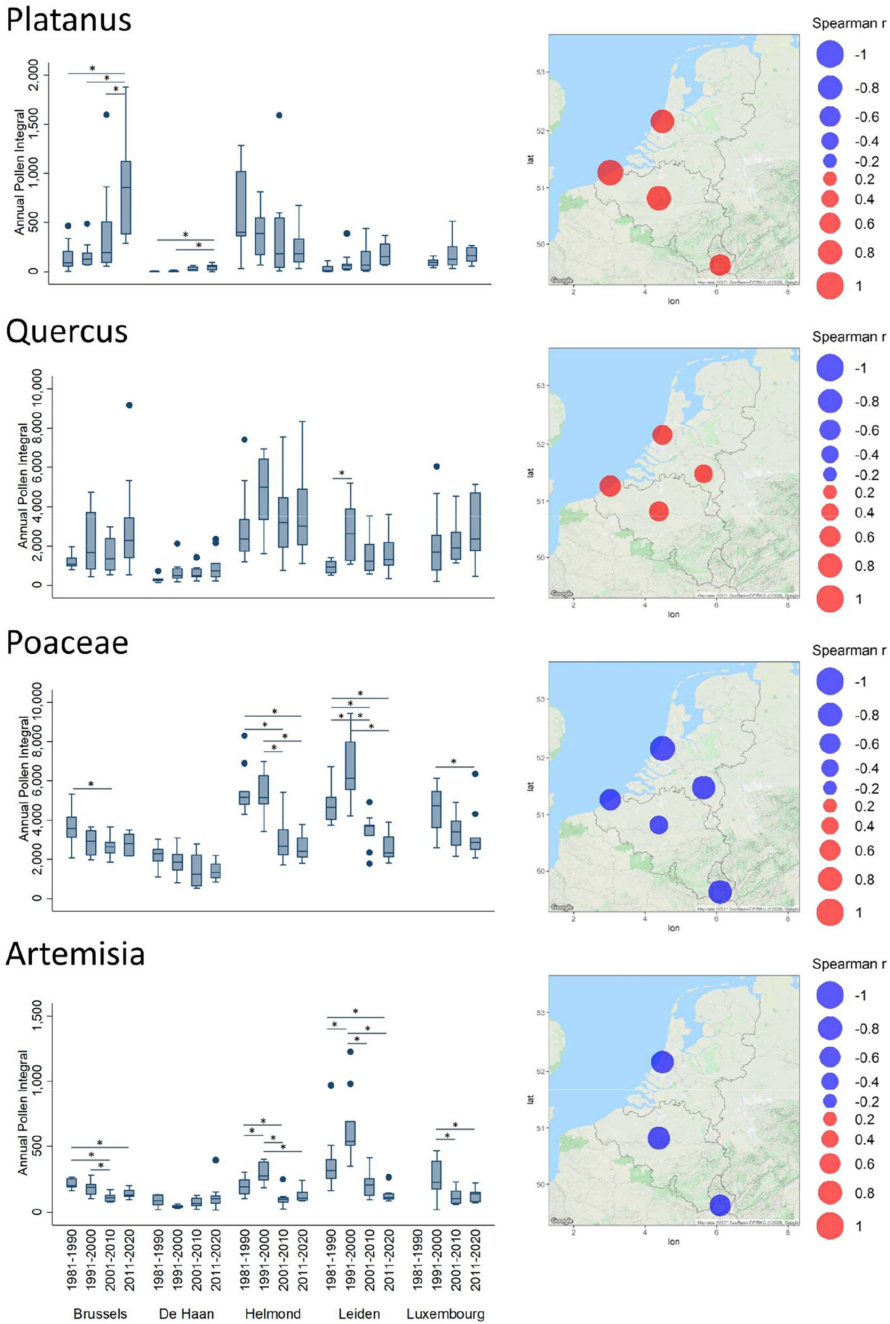
Trends of Annual Pollen Integrals (API) for *Platanus, Quercus*, Poaceae, and *Artemisia*. For further details see, legend of Figure 2.

Results are more contrasting for *Platanu*s pollen (Figure 3), with API values that tend to increase in Brussels, De Haan, Leiden, and Luxembourg, while they seem to tend to decrease weakly in Helmond. In Brussels, the decadal increase was significant for each selected decade (Figure 3).

Among the last blooming allergenic trees are the oaks, which have most likely been tending to emit and disperse more pollen over the decades, as shown by significantly positive Spearman correlation values (Supplementary Table 2) in Brussels, De Haan, Leiden, and Helmond (not significant in Luxembourg). As for *Alnus* there is a significant increase in annual airborne pollen in the decade 1991–2000 as compared to the other decades in Leiden and also in Helmond (Figure 3). Finally, the long-term trends of the season intensity for herbaceous plants (Figure 2 and Supplementary Table 2) show a decrease in pollen amounts, in contrast to all the studied tree taxa. In particular, grass API has significantly been decreasing in all Benelux stations. After a temporary rise in the decade 1991–2001 a significant decrease is noticeable in the following decades 2000–2010 in Leiden and Helmond. Also, in Luxembourg a significant decrease between 1991–2000 and 2001–2020 was observed. A similar pattern exists in Leiden and Helmond for *Artemisia* with a significant increase in API during the decade 1991–2000 (Figure 3). The Spearman correlation (Supplementary Table 2) showed significantly decreasing *Artemisia* APIs in Leiden, Brussels, and Luxembourg. However, exposure to this allergenic pollen taxon has always been low in this region with respect to absolute values.

**Figure 4:**
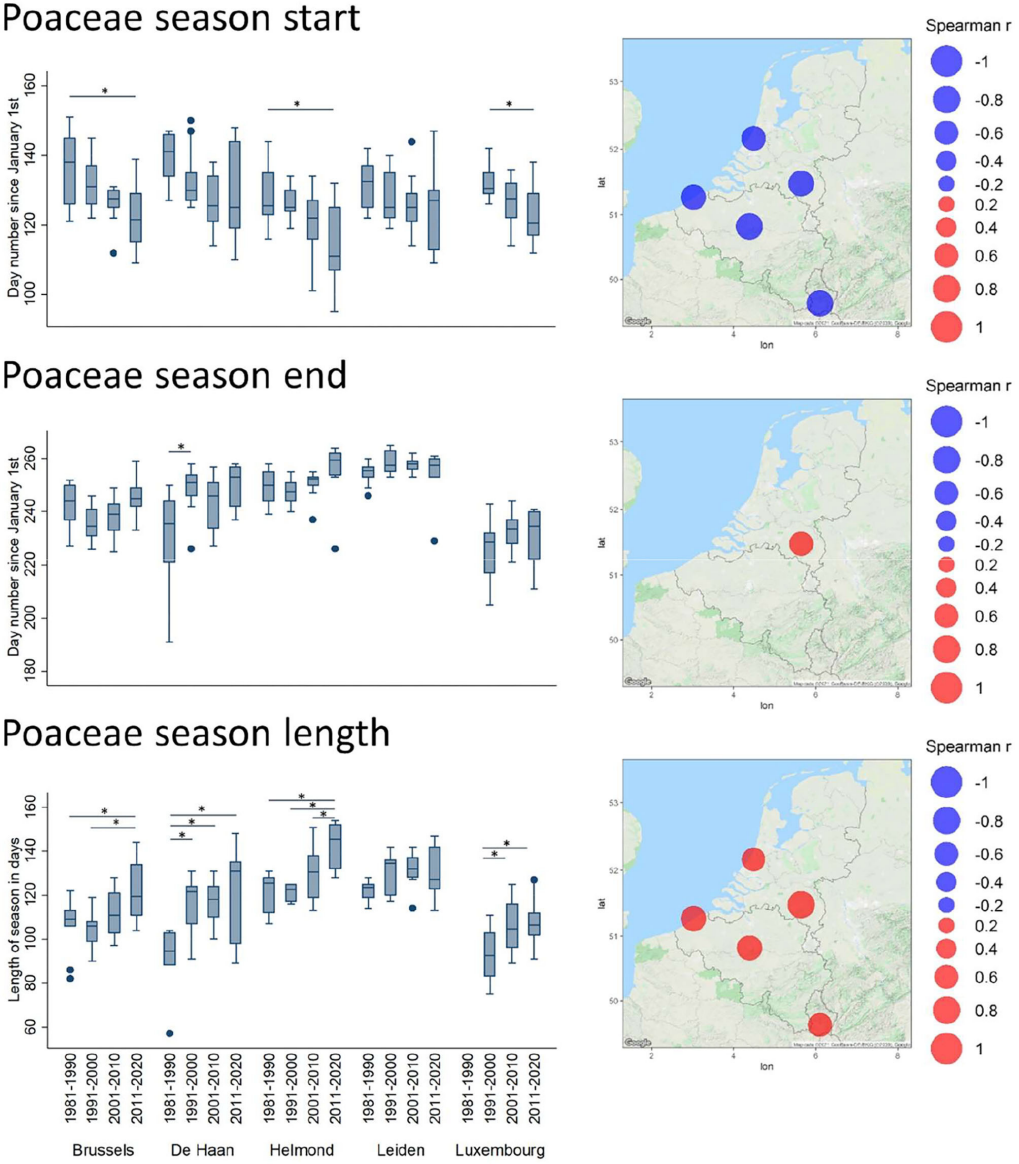
Trends of timing shifts in pollen seasons for *Quercus*. Spearman correlation coefficient (Rho) values between the day of the year for the season start/end and years, and between season length and years, are plotted in bubble maps at the location of the monitoring stations, from negative (in blue) to positive (in red). The bubble size relates to the magnitude of the Rho value. Non-significant values are not shown on the maps. Timing parameters, averaged per decade (1981–1990, 1991–2000, 2001–2010, and 2011–2020), are depicted in boxplots. For further details, see legend of Figure 2.

**Figure 5:**
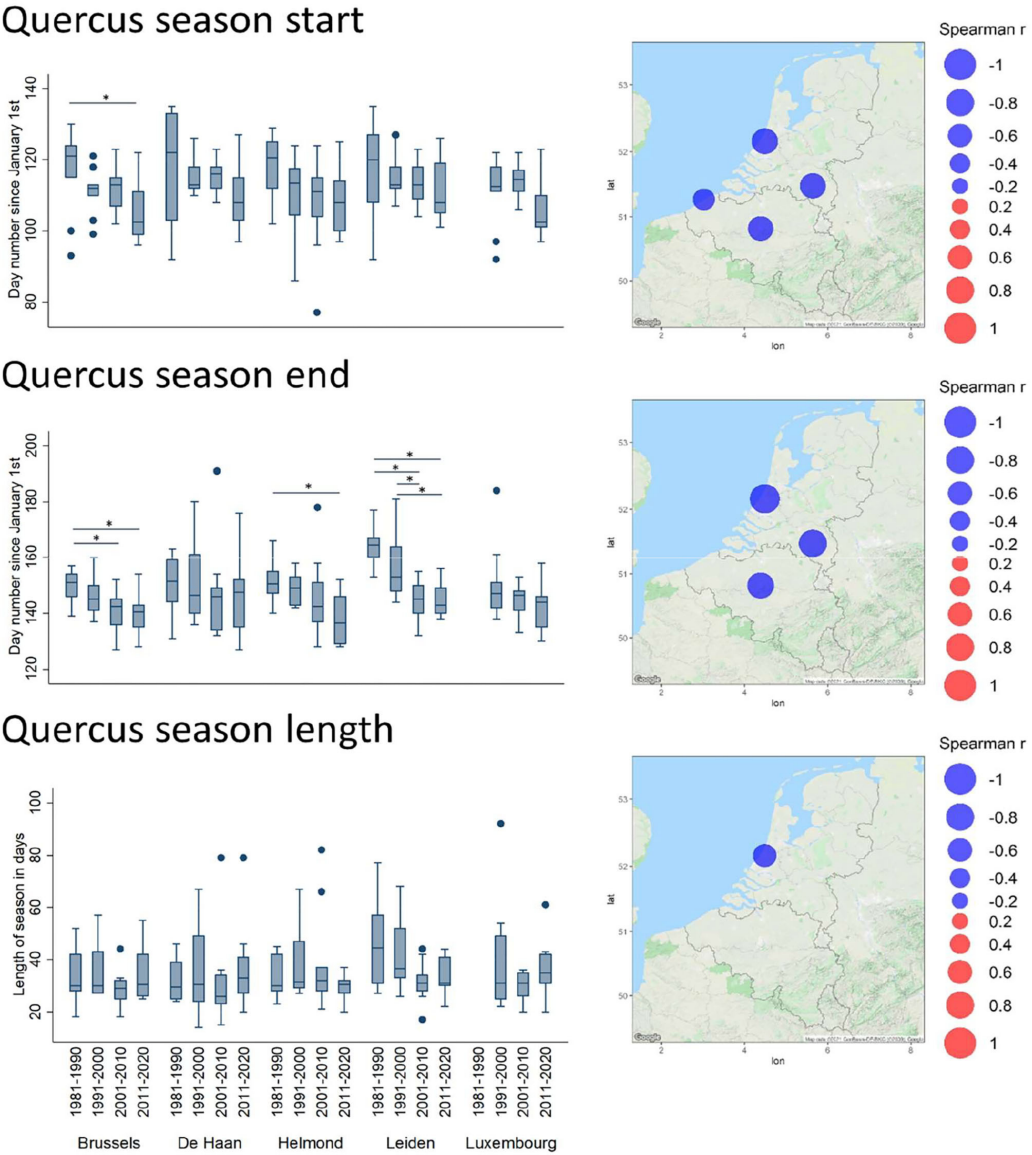
Trends of timing shifts in pollen seasons for Poaceae. For further details see, legend of Figure 4.

In terms of timing, the Spearman correlation analysis of the annual parameters revealed several significant temporal shifts. In all significant cases, the seasonal start has shifted toward earlier dates in the year (Supplementary Table 2). The most remarkable are those of the oak and grass pollen season (Supplementary Table 2 and Figure 4). Indeed, for *Quercus*, this shift was characterized by a significantly earlier season start date (1% API) in all stations (excepting Luxembourg), and by a significantly earlier seasonal end date (99% API) in all stations (excepting Luxembourg and De Haan). The decade plots show trends toward an earlier start and end of the pollen season with significant decadal changes in the end of the season for Brussels Helmond and Leiden (Figure 4). Overall, this shift has not involved a shortening of the seasonal length, except for Leiden that shows a low but significant shortening trend of the length of the season. The Poaceae season, which slightly overlaps with the end of the oak season, also appears to progressively start earlier over the decades in all stations (Figure 5). In contrast, this earlier season start is not coupled to an earlier seasonal end, which has a tendency to be later in Helmond. Therefore, the resulting trend in grass pollen season length has clearly tended to increase in all stations, as shown by significantly positive Spearman correlation values in all stations and also by the significant decadal changes in length for all stations except Leiden (Supplementary Table 2 and Figure 5).

Besides, it is interesting to observe a series of changes for specific timing parameters of some taxa. For instance, the pollen season of *Corylus*, which is already among the earliest of the year, has tended to start even earlier over the decades. This is shown by significantly negative correlation values between season start dates (1% API) and years, in all stations except in Luxembourg (Supplementary Table 2). The same significant trend is observed for the start of *Fraxinus* pollen season, which has also progressively started earlier in the year, i.e., in Brussels, De Haan, and Helmond. Finally, significantly negative trends are observed in all stations regarding the end of the *Betula* pollen season.

The statistical association between the temporal pollen and weather trends measured in all the Benelux stations—which is an indication of climate change effects on pollen releases—suggests a substantial contribution of the increasing temperature and/or radiation on the increasing amounts of pollen emitted by birch trees during the spring (Table 1). The changes we observe over time for the *Alnus* season (flowering in winter) and Poaceae season (flowering in summer) were more complex than those of the *Betula* season, both temporally (different stages of the season are altered in different locations) and geographically (significant associations with some weather variables in some locations but not others). The analysis of the association between *Alnus*, or Poaceae pollen and weather trends was thus inconclusive in the sense that we did not observe a uniform or generalizable pattern.

**Table 1:** Association between the rates of change in the seasonal cycles for birch pollen concentrations and the corresponding temperature and radiation change rates.

**Station**	**Temperature**			**Radiation**
	**Mean**	**Maximum**	**Minimum**	
Brussels	0.72^***^	0.86^***^	0.90^***^	−0.09 ns
De Haan	0.09 ns	0.28 ns	0.00 ns	0.79^***^
Helmond	0.19 ns	0.18 ns	0.27^*^	0.33^*^
Leiden	0.63^***^	0.68^***^	0.59^***^	0.78^***^
Luxembourg	0.79^***^	0.82^***^	0.80^***^	0.21 ns

* *P < 0.05*;

*** *P < 0.001; ns, not significant*.

*Spearman correlation coefficient (Rho) was computed between the daily Sen slopes of the birch pollen levels and the daily Sen slopes of the corresponding temperature and radiation*.

## Discussion

The results show that airborne pollen concentrations are subject to complex dynamics mainly depending on the type of pollen and to a relatively lesser extent on the location. Indeed, distanced by a maximum of 300 km (from Leiden to Luxembourg City), we observe similar trends in API, season start, end and length both upward and downward, for all aerobiological stations in the Benelux, which is in line with previous findings (16, 35). Given the biogeographic region gradient from NW to SE, one might have expected more latitude dependent discrepancies. Certainly, some differences are noticeable, such as the slightly less pronounced trend of increase in intensity for the Betulaceae tree family in the North of the Benelux. But this is probably partly due to the fact that some species such as alder and birch are already well-established in this Northern region. Some recent studies from Central Europe (34, 36) also showed increases in pollen load and advances in the start of the season for Betulaceae species, which seem to be driven by climate change. Another, multi-site study performed in the Northern hemisphere (35) showed that the impact of temperature on the increase in pollen concentrations, in terms of intensity and duration, appears to be global and independent of latitude. Our results are in line with these findings.

The analysis of the *Betula* pollen change rate and the corresponding change rate in temperature and radiation showed significantly positive correlation at three of the five sites (Table 1). The *Betula* pollen season is relatively short consisting mostly of one or two major peaks and the birch trees typically flower during late March and April, a period which shows the highest positive changes in the Sen slopes of temperature and radiation over decades at these study sites (data not shown). Considering temperature changes it need to be noticed that the study sites are located in urban environments and may also be influenced by urban heat island effects (16).

The lack of statistically significant associations does not exclude the possibility that additional weather variables contribute to the observed changes, only that their effect is below the sensitivity of our analysis. For instance, precipitation can have opposing effects, reducing pollen release in the short-term through wet deposition, while potentially increasing pollen production as a longer-term effect due to its impact on plant water availability. Such complexity makes the detection of an effect more difficult. For other taxa such as Poaceae and *Alnus*, the analysis of association was inconclusive in that we did not identify a uniform or generalizable pattern in the five locations. It might be hampered due to the longer flowering periods and the existence of multiple successive flowering (due to early and late flowering species). And particularly for grasses, their long pollen season covers very different meteorological patterns from late spring to summer. The relationship between weather trends and pollen trends in the context of climate change is complex and warrants further study. Furthered, it is assumed that climate change indirectly impacts airborne pollen concentrations by disrupting their production cycle (also called mast seeding cycle), resulting in an increase in the frequency of pollen-rich years, coupled with an average increase in seasonal amounts (37, 38). It should also be noted that the trend toward an increase in the intensity of the *Fraxinus* season in Luxembourg is masked (non-significant correlation coefficient) because of this high variance, showing the importance of analyzing trends using several complementary methods.

In addition, as a more general reminder, if we aim to increase the quality of such trend analyses in the future, it is essential to guarantee the continuity of monitoring over the long term. Indeed, statistical tests will in principle be more efficient in highlighting a significant trend if the time series are longer. The most recent years of observation are therefore beginning to remarkably complete the oldest data series and to highlight new trends that were previously undetectable. For example, the significantly earlier start of the hazel pollen season in Brussels was shown in this study (data up to 2020) whereas in a previous study (17), it was not significant (data up to 2015). Also, the decreasing trends for Poaceae pollen observed in the current study was not detected in a previous study with data from Leiden and Brussels up to 2001 (39).

From the results of this study, we also note that, regardless of variance, the API averaged per decade could in some cases show varying rates of change from one decade to the next. The best example is the variation of API in Leiden and Helmond for the pollen of herbaceous plants (Poaceae and *Artemisia*). The increase in API during the 1991–2000 decade is most likely caused by the waste/fallow land in the close vicinity of both monitoring stations in Helmond and Leiden in that period. In subsequent years, those areas were rebuilt which resulted in a reduction in the number of Poaceous and *Artemisia* plants in the neighborhood of the sites and concomitantly a reduction in API of those species is observed in those subsequent decades. Previous studies also indicate that increasing levels of urbanization result in lower grass pollen concentrations (40, 41), which would explain why the trend in herbaceous plant pollen concentrations is generally downward in the largest cities of this study. In the case of grasses, this decrease in intensity is accompanied by a longer season length (due to a progressively earlier start of the season). Longer pollen season in urban environments has also been reported by others (40, 42). However, it might be that there is also an effect of climate on the flowering of certain grass species of this large family, which is very sensitive to temperature and water availability (13, 43). This paradoxical observation is also similar to that reported in Brussels using another method (17), where the season duration defined by the number of days >1 pollen/m3 tended to increase while the duration defined by the number of days >50 pollen/m3 tended to decrease. It is therefore tempting to extrapolate this with the results of our study, by interpreting that the symptomatic season (the so-called high season) for grass pollen has progressively been decreasing in the whole Benelux. Definitely, this needs more research.

To conclude, our results show that temporal variations in annual pollen levels almost always follow a similar trend in the Benelux, suggesting the impact of large-scale climate change-driven factors, especially for birch pollen. Trends in herbaceous species tend to be more local-specific, most likely under the influence of other environmental factors, e.g., the increasing urbanization in the surroundings of these monitoring locations. In all, these aeroallergen exposure dynamics might have direct impacts on public health, either by increasing the severity of the allergic symptoms, or by requesting an adjustment in treatment timing, or by complicating specific allergy diagnosis if pollen seasons overlap, and by the occurrence of new types of pollen induced respiratory allergies that have so far a weak prevalence in the Benelux, e.g., against plane and ash pollen.

## Data Availability Statement

The data can be made available upon request to the corresponding author.

## Author Contributions

Analysis was carried out by LW, NB, WV, AD, and PH. LW and NB drafted the first version of the manuscript. All authors contributed to revision and final approval of the manuscript and provided contributions to study conception and design and acquisition of data and interpretation of the results.

## Conflict of Interest

The authors declare that the research was conducted in the absence of any commercial or financial relationships that could be construed as a potential conflict of interest.
