# Supplementary material for: Long-Term Pollen Monitoring in the Benelux: Evaluation of Allergenic Pollen Levels and Temporal Variations of Pollen Seasons
Source: Front Allergy. 2021 Jul 8;2:676176. doi: 10.3389/falgy.2021.676176 (PMC8974733; doi:10.3389/falgy.2021.676176)
Supplement: Supplementary file 1 [file Data_Sheet_1.PDF]

Supplementary table 1: Overview of the study years used in the analysis for the 5 sites. Any major data gap during the usual window of a specific pollen season resulted in the omission of the related yearly dataset for a given taxon.

	Brussels		De Haan		Leiden		Helmond		Luxembourg	
	period of data collection	No of years	period of data collection	No of years	period of data collection	No of years	period of data collection	No of years	period of data collection	No of years
data available	1982-2020	39	1984 - 2020	37	1977-2020	44	1977-2020	44	1991-2020	30
	missing years									
Alnus	-	39	1987,1988	35	-	44	1977-1990	30	1991-1999 2003	20
Corylus	-	39	1987,1988, 1990	34	-	44	1977-1990	30	1991-1999 2003	20
Betula	-	39	2007,2012	35	-	44	1977-1981 1990	38	-	30
Fraxinus	-	39	2007,2012	35	-	44	-	44	-	30
Quercus	-	39	1988, 2009	35	-	44	1998; 2000	42	-	30
Platanus	-	39	-	37	1977-1982	39	1977-1986; 1988:2000	33	1991	29
Poaceae	-	39	2014, 2019, 2020	34	2009	43	1991;1998; 1999;2000	40	-	30
Artemisia	-	39	1988, 2008	35	1994;2009	42	1979;1988; 1989;1991; 2000	39	1992;1994	28

Supplementary table 2: Spearman correlation coefficient (Rho) values between season parameters and years. All significant values are in bold**.** When p<0.05, values are colored in dark red (if positive) or blue (if negative). When p<0.1, values are colored in light red (if positive) or light blue (if negative). Start1%, End99% and Length1 are based on the 1% and 99% of the SPI definition. Start5%, End95% and Length5 are based on the 5% and 95% of the API definition. The * indicates missing parameters related to the timing of the season due to too low API numbers, which hamper the reliable calculation of these parameters.

		Number of pollen			Season characteristics								
ALNUS		API	Peak		Peakday		Start1%	end99%	length1		Start5%	end95%	length5
Belgium	Brussels	**0,477**	**0,386**		**-0,400**		-0,193	-0,049	0,027		-0,157	-0,139	0,005
	Den Haan	**0,599**	**0,338**		**-0,380**		**-0,508**	0,067	**0,575**		**-0,400**	-0,159	**0,404**
Netherlands	Helmond	-0,011	0,021		-0,098		-0,227	**-0,312**	0,259		0,278	0,133	-0,193
	Leiden	**0,306**	**0,262**		-0,126		**-0,490**	**-0,371**	0,098		**-0,407**	**-0,304**	**0,285**
Luxemburg	Luxembourg	**0,543**	**0,478**		0,264		-0,216	-0,036	0,160		0,022	0,133	0,091
BETULA													
Belgium	Brussels	**0,418**	**0,394**		**-0,463**		**-0,302**	**-0,467**	-0,103		-0,246	**-0,385**	-0,043
	Den Haan	**0,281**	0,184		**-0,303**		-0,188	**-0,323**	-0,111		-0,245	-0,235	0,027
Netherlands	Helmond	-0,044	0,005		**-0,324**		-0,265	**-0,466**	-0,080		**-0,371**	**-0,313**	0,174
	Leiden	0,204	0,221		**-0,485**		-0,104	**-0,641**	**-0,506**		**-0,358**	**-0,563**	**-0,296**
Luxembourg	Luxembourg	**0,366**	0,301		-0,282		0,009	**-0,486**	**-0,431**		-0,125	**-0,309**	-0,154
CORYLUS													
Belgium	Brussels	**0,622**	**0,538**		-0,192		**-0,415**	0,127	**0,349**		**-0,341**	-0,105	0,204
	Den Haan	**0,640**	**0,504**		**-0,499**		**-0,627**	-0,054	**0,424**		**-0,439**	-0,264	0,216
Netherlands	Helmond	0,182	0,037		-0,289		**-0,628**	-0,481	0,022		-0,338	0,427	-0,072
	Leiden	0,221	0,056		**-0,390**		-0,138	-0,151	0,014		-0,207	-0,121	0,112
Luxembourg	Luxembourg	**0,384**	0,338		0,128		-0,152	0,075	0,270		-0,167	0,000	0,301
FRAXINUS													
Belgium	Brussels	**0,336**	0,212		**-0,269**		**-0,358**	**-0,450**	0,139		**-0,315**	**-0,466**	0,044
	Den Haan	**0,572**	**0,394**		-0,120		**-0,468**	0,104	**0,679**		**-0,369**	-0,025	**0,558**
Netherlands	Helmond	**0,593**	**0,481**		**-0,583**		**-0,305**	**-0,536**	-0,022		-0,230	**-0,530**	-0,230
	Leiden	**0,381**	**0,328**		-0,097		0,240	**-0,606**	**-0,434**		0,207	**-0,455**	**-0,487**
Luxembourg	Luxembourg	0,245	0,174		0,039		-0,297	**-0,312**	0,048		-0,231	-0,301	-0,017
QUERCUS													
Belgium	Brussels	**0,356**	0,252		**-0,439**		**-0,470**	**-0,549**	-0,023		**-0,570**	**-0,590**	0,142
	Den Haan	**0,456**	**0,410**		**-0,526**		**-0,350**	-0,185	0,056		**-0,465**	**-0,378**	0,104
Netherlands	Helmond	**0,292**	0,186		**-0,645**		**-0,496**	**-0,650**	-0,060		**-0,611**	**-0,710**	-0,014
	Leiden	**0,350**	**0,400**		**-0,629**		**-0,515**	**-0,780**	**-0,361**		**-0,606**	**-0,695**	-0,019
Luxembourg	Luxembourg	0,227	0,140		**-0,463**		-0,277	-0,234	0,102		**-0,320**	-0,236	0,068
PLATANUS													
Belgium	Brussels	**0,684**	**0,675**		**-0,506**		**-0,342**	**-0,414**	-0,036		**-0,391**	**-0,615**	-0,202
	Den Haan												
Netherlands	Helmond	-0,223	-0,123		**-0,328**		-0,184	**-0,518**	-0,271		-0,154	**-0,511**	-0,261
	Leiden	**0,586**	**0,610**		**-0,447**		**-0,395**	-0,258	0,139		**-0,406**	**-0,520**	-0,114
Luxembourg	Luxembourg	**0,438**	**0,575**		0,005		-0,066	-0,054	0,036		0,034	-0,242	-0,208
POACEAE													
Belgium	Brussels	**-0,287**	-0,249		**-0,406**		**-0,557**	0,167	**0,463**		**-0,430**	**-0,290**	0,151
	Den Haan	**-0,435**	-0,253		-0,254		**-0,592**	**0,374**	**0,598**		**-0,458**	0,118	**0,452**
Netherlands	Helmond	**-0,597**	**-0,538**		-0,182		**-0,526**	**0,417**	**0,606**		**-0,655**	0,162	0,519
	Leiden	**-0,709**	**-0,479**		**-0,316**		**-0,437**	0,115	**0,382**		-0,251	-0,150	0,011
Luxembourg	Luxembourg	**-0,602**	-0,303		**-0,470**		**-0,551**	0,139	**0,395**		**-0,448**	-0,128	0,202
ARTEMISIA													
Belgium	Brussels	**-0,527**	**-0,358**		-0,255		-0,062	0,127	0,159		**-0,289**	0,065	**0,365**
	Den Haan	0,044	0,100		**-0,402**		**-0,280**	**0,380**	**0,475**		-0,244	**0,359**	**0,465**
Netherlands	Helmond	-0,284	**-0,413**		**-0,384**		-0,088	0,264	0,211		-0,231	0,176	0,249
	Leiden	**-0,512**	**-0,544**		**-0,266**		**-0,543**	0,133	**0,485**		**-0,544**	-0,021	**0,422**
Luxembourg	Luxembourg	**-0,440**	**-0,395**		**-0,411**		0,143	0,067	0,014		-0,129	-0,245	-0,084
